# Supplementary material for: Identification of Missense Variants Affecting Carcass Traits for Hanwoo Precision Breeding
Source: Genes (Basel). 2023 Sep 22;14(10):1839. doi: 10.3390/genes14101839 (PMC10606632; doi:10.3390/genes14101839)
Supplement: Supplementary file 1 [file genes-14-01839-s001.zip › Figure S2. Multiple sequence alignment and protein structure prediction models.pdf]

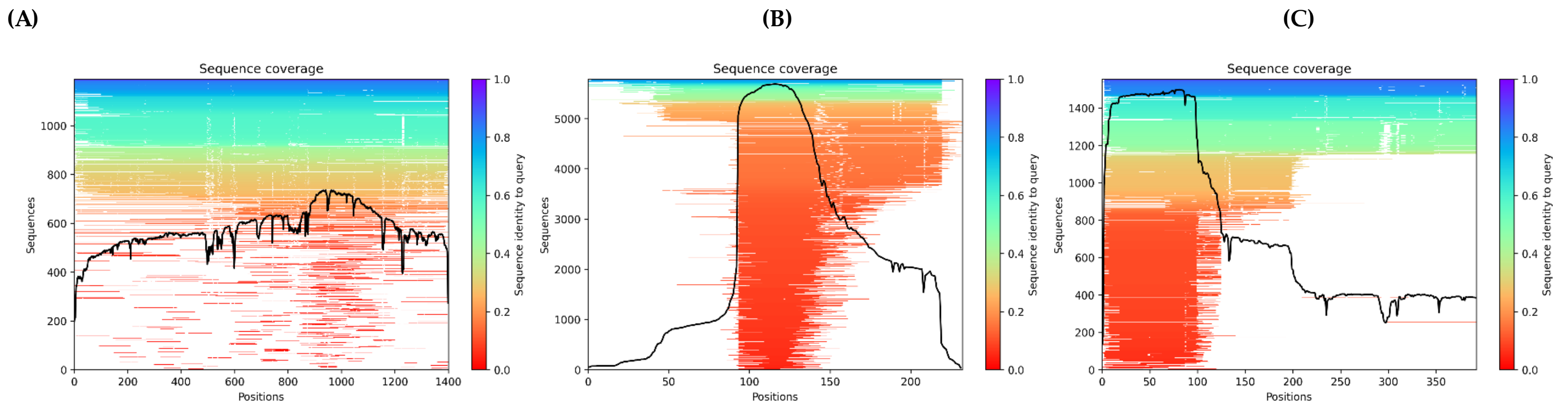

Figure S2. Multiple sequence alignment (MSA) on carcass traits.

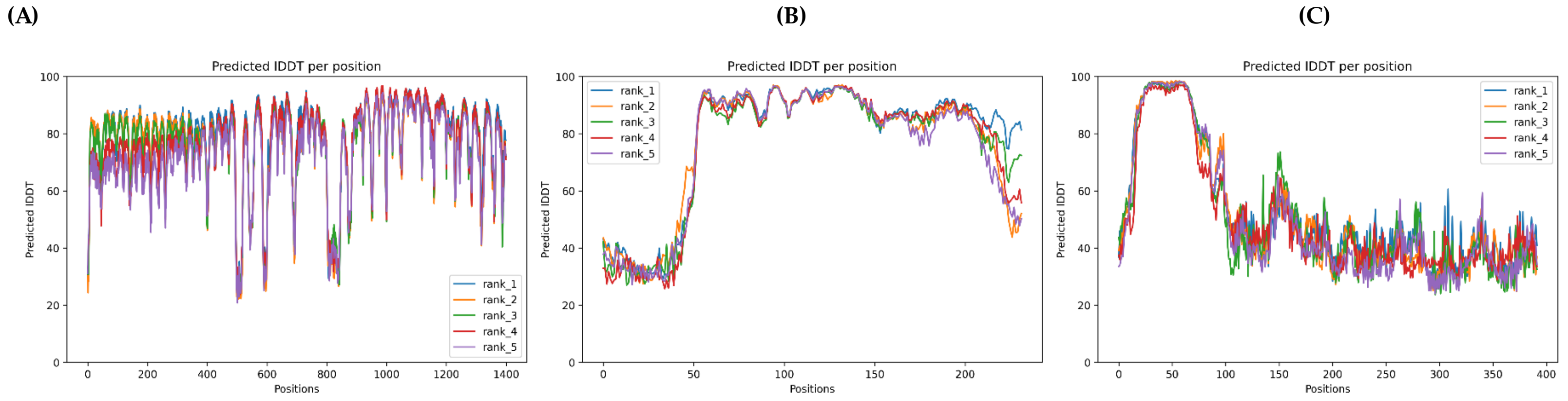

Figure S3. Predicted local distance difference test (pLDDT) score per position for 5 models.

(A)

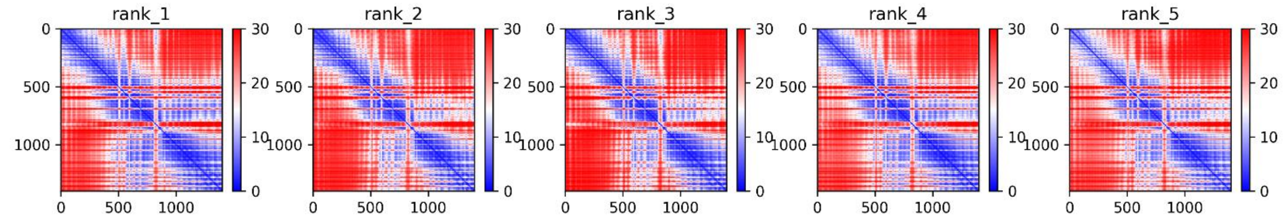

(B)

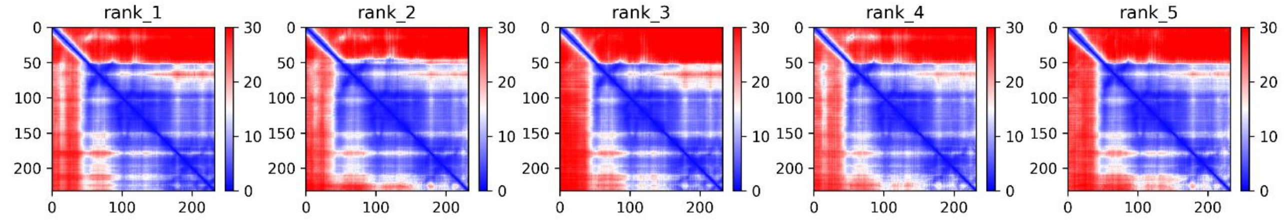

(C)

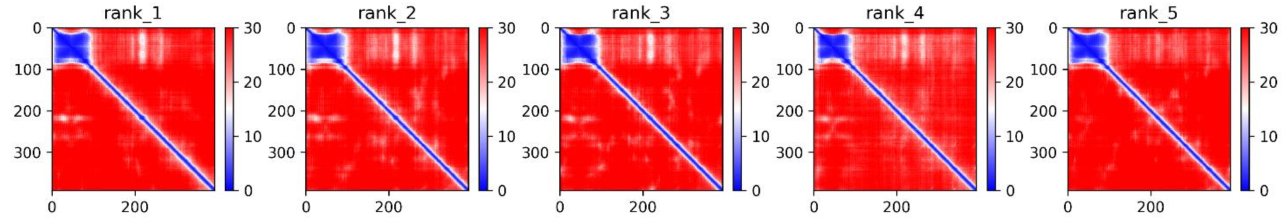

Figure S4. Predicted aligned error (PAE) score for five predicted models.
