# Supplementary figures and images for: Identification of Missense Variants Affecting Carcass Traits for Hanwoo Precision Breeding
Source: Genes (Basel). 2023 Sep 22;14(10):1839. doi: 10.3390/genes14101839 (PMC10606632; doi:10.3390/genes14101839)

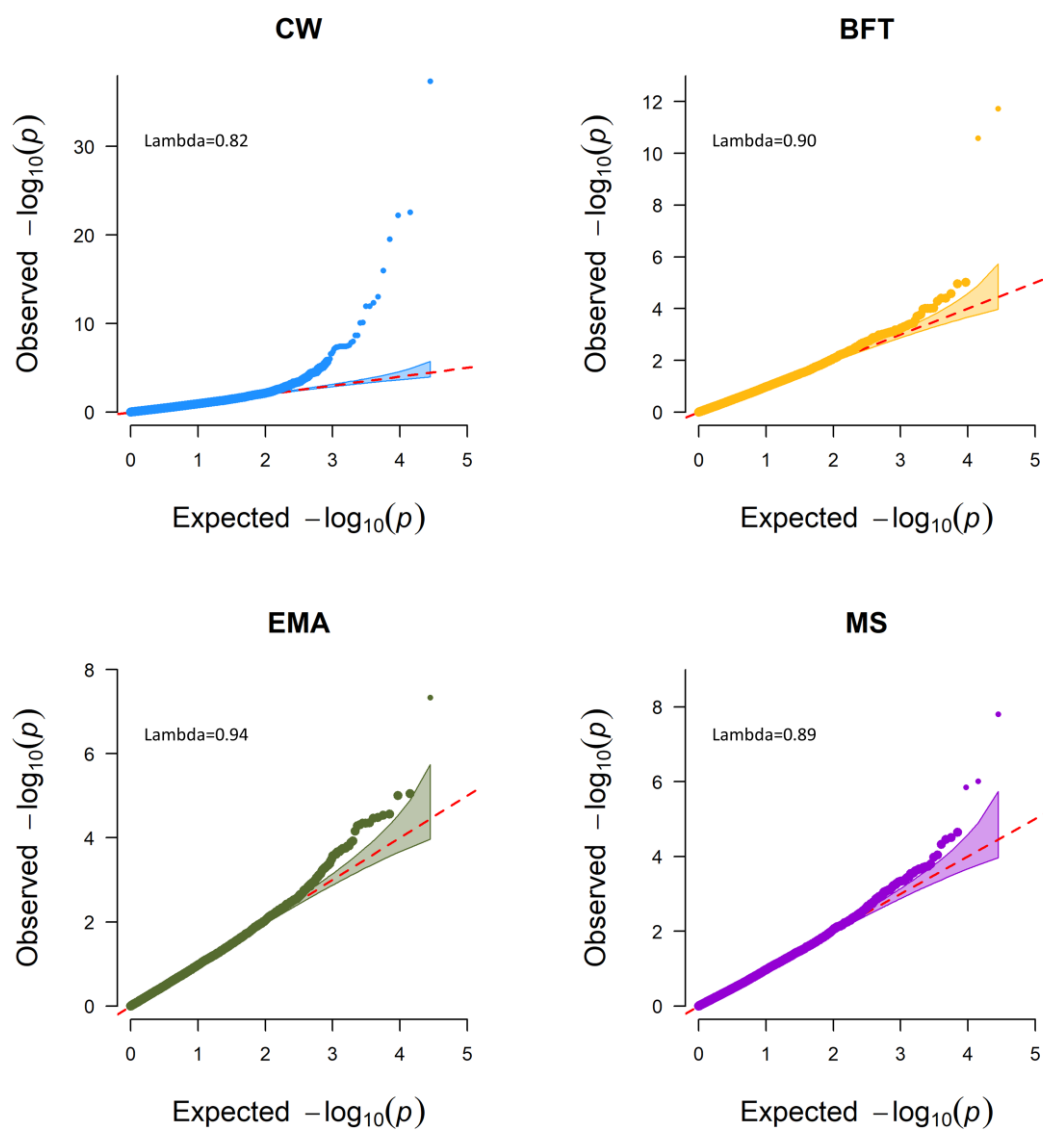

**Figure S1.** QQ plot of exon-specific association studies on carcass traits.

Supplement: Supplementary file 1 [file genes-14-01839-s001.zip › Figure S1. QQ plot of exon-specific association studies on carcass traits.pdf]
